# Beyond antibody engineering: directed evolution of alternative binding scaffolds and enzymes using yeast surface display

**DOI:** 10.1186/s12934-018-0881-3

**Published:** 2018-02-26

**Authors:** Doreen Könning, Harald Kolmar

**Affiliations:** 1 Antibody-Drug Conjugates and Targeted NBE Therapeutics, Merck KGaA, Frankfurter Strasse 250, 64293 Darmstadt, Germany; 2 Institute for Organic Chemistry and Biochemistry, Technische Universität Darmstadt, Alarich-Weiss-Strasse 4, 64287 Darmstadt, Germany

**Keywords:** Yeast surface display, Scaffold protein, Enzyme, Alternative scaffold protein

## Abstract

Pioneered exactly 20 years ago, yeast surface display (YSD) continues to take a major role in protein engineering among the high-throughput display methodologies that have been developed to date. The classical yeast display technology relies on tethering an engineered protein to the cell wall by genetic fusion to one subunit of a dimeric yeast-mating agglutination receptor complex. This method enables an efficient genotype–phenotype linkage while exploiting the benefits of a eukaryotic expression machinery. Over the past two decades, a plethora of protein engineering efforts encompassing conventional antibody Fab and scFv fragments have been reported. In this review, we will focus on the versatility of YSD beyond conventional antibody engineering and, instead, place the focus on alternative scaffold proteins and enzymes which have successfully been tailored for purpose with regard to improving binding, activity or specificity.

## Background

Directed evolution is a powerful method that involves (1) the random generation of a broad set of protein variants, (2) their production in an expression host, and (3) the subsequent screening for variants with desired novel functionalities [1–3]. The method was enabled by the emergence of cell surface display techniques that bring proteins of interest into direct contact with potential interaction partners. Notably, the yeast *Saccharomyces cerevisiae* proved to be an invaluable tool for the generation of large protein libraries, where each variant is displayed in high copy number on the surface of a single cell, thereby converting gene diversity into cell diversity. Ultra-high throughput yeast library screening has been extensively used in pharma and biotech industry for the screening of large antibody repertoires aimed at isolating variants with therapeutic relevance. This review focuses on directed evolution of alternative scaffolds and enzymes engineered to improved target binding, specificity or activity using yeast surface display. The versatility of this screening platform will be emphasized by describing many examples of the engineering of non-antibody molecules as well as functional screening strategies for the modification of enzymes.

## Introduction

The expression and display of proteins on the surface of bacterial and eukaryotic host cells has become increasingly attractive, as demonstrated by the numerous platform technologies that have been developed [4–8]. In contrast to bacteria, for which potent display methods have been established over the years [9], eukaryotes offer the additional advantage of an efficient posttranslational modification machinery as well as a quality control mechanism for protein folding that encompasses chaperones and foldases [10]. In particular, surface display on *S. cerevisiae* has emerged as a powerful tool for the isolation of binding molecules with achievements of library sizes up to 10^9^ transformants [11]. Since strategies for the generation and high-throughput screening of large combinatorial libraries of human antibodies using yeast surface display have been extensively reviewed elsewhere [12], the focus of this review is placed on the isolation of tailor-made binding proteins as well as enzymes with improved functional characteristics. This review highlights the versatility of the yeast surface display platform beyond classical antibody engineering and provides an overview of the many engineering approaches that have successfully been conducted with regard to improving not only protein binding but also enzyme activity and specificity.

## Surface display on *Saccharomyces cerevisiae*

**Fig. 1:**
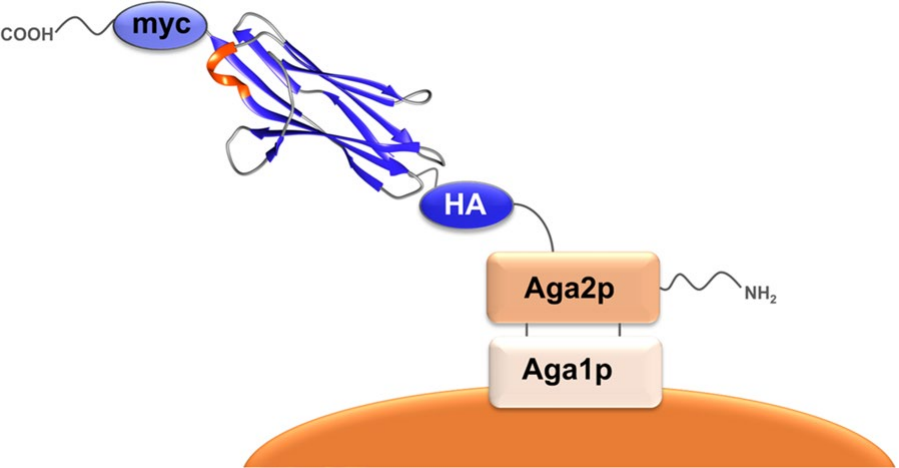
Yeast surface display setup as pioneered by Boder and Wittrup in 1997 [1]. The protein of interest (a vNAR domain in this particular depiction) is fused to the *C*-terminus of the Aga2p protein. Aga2p is covalently linked to Aga1p via two disulfide bonds. Aga1p anchors the fusion protein to the cell wall, ensuring a genotype–phenotype coupling of individual yeast cells. The vNAR structure has been modified from pdb identifier 4GHK using UCSF Chimera [133]

In general, the principle of microbial cell surface display relies on the establishment of a genotype–phenotype linkage which converts gene diversity into protein diversity. This link is an essential pre-requisite for the success of any surface display screening platform and it is usually realized upon fusing the protein of interest to a microbial cell surface protein. In the case of yeast surface display, a variety of different anchor proteins has been evaluated for efficiently tethering the protein of interest to the cell wall [10]. The most commonly used anchor is the *S. cerevisiae a*-agglutinin mating complex that consists of two subunits referred to as Aga1p and Aga2p. The classical yeast surface display method as pioneered by Boder and Wittrup in [4] relies on the *N*-terminal fusion of a protein of interest to Aga2p (Fig. 1). Nevertheless, the orientation can be altered upon employing *C*-terminal fusions, depending on the protein to be displayed, as for some proteins a free *N*-terminus can be crucial for efficient functionality [13]. Depending on the cell wall protein that is utilized for immobilization, the number of copies of the protein of interest that are displayed can vary [14]. When using the Aga2p system, however, it has been demonstrated that up to 10^5^ copies of the fusion protein can be displayed on a single cell [4]. For some proteins, surface display efficiency correlated with protein secretion levels, i.e. proteins with favorable mutations resulting in increased thermal stability or protein folding could be presented at a higher copy number in contrast to the respective wildtype [15–18]. For instance, Kieke and coworkers achieved good surface display levels for a previously display-incompetent single-chain T cell receptor. Upon combining several stability-enhancing mutations, they improved the display levels from 10,000 up to 50,000 copies per yeast cell [15].

Genetically, the Aga2p fusion protein is encoded on a plasmid and its expression is under the control of a galactose-inducible promotor (GAL1). The Aga1p protein on the other hand is encoded in the yeast genome and also controlled by a GAL1 promotor sequence. The assembly of Aga1p and Aga2p is ensured by the formation of two disulfide bonds. For subsequent functional screenings, Boder and Wittrup included epitope tags which were fused to the *C*-terminus of the protein of interest or inserted between the Aga2p and the protein of interest. Upon immunofluorescence staining of these tags, full-length protein expression can be verified using a flow cytometer. This offers an additional quality control check during the isolation of variants with desired functionalities and represents a distinct advantage over phage display [19, 20]. However, detection of a *C*-terminal tag does not give information on the structural integrity of the displayed protein. This obstacle can be circumvented upon using a conformation-specific detection antibody for the protein of interest [21]. After the incubation of yeast cells with the respective target protein, the interaction can be analyzed using fluorescently-labeled detection reagents specifically addressing the target.

The generation of yeast libraries for the purpose of identifying a protein variant with superior abilities such as improved stability, affinity or, in case of enzymes, higher catalytic activity usually relies on the mutagenesis of a precursor protein. Mutations can be introduced through error-prone PCR [22, 23], DNA-shuffling [24, 25], codon-based randomization [26, 27] or structure-guided design [28]. Subsequently, yeast cells are transformed with the genetic library resulting in genotype–phenotype linked yeast libraries with sizes up to 10^9^ transformants. Although several orders of magnitude smaller than libraries generated using phage, ribosomal or mRNA display, the utilization of yeast display offers the inherent advantage of simultaneously analyzing the library content in terms of surface display (via the detection of epitope tags) and target binding, thereby enabling a functional read-out.

### Alternative binding proteins and yeast surface display

Yeast surface display has emerged as a straightforward strategy for human antibody engineering. This topic has been extensively reviewed and, therefore, will not be highlighted herein [10, 12, 29, 30]. Beyond antibodies, alternative scaffold-based affinity reagents have emerged as a promising class of biomolecules with therapeutic potential [31–38]. These proteins exhibit advantageous properties in comparison to full-length monoclonal antibodies, such as improved tissue penetration, superior stability and cost-efficient production [32, 39]. In general, an alternative scaffold protein is capable of exhibiting a variety of amino acid sequences on a constant backbone region [40]. A pre-requisite that renders a protein an ideal alternative binding scaffold is a certain tolerance towards structural alterations which are necessary in order to tailor the protein for purpose and enable molecular recognition [41, 42]. In contrast to conventional antibodies, they are often able to interact with cryptic or hidden epitopes that are difficult to address. As an example, shark-derived vNAR domains as well as camelid-derived VHH domains have been reported to specifically engage the cleft-like catalytic site of enzymes [43–45]. In addition, recombinant production of these scaffolds is often cheaper compared to the costs of producing monoclonal antibodies, as no posttranslational modifications are required and recombinant expression in *Escherichia coli* rather than in mammalian cells can be performed. Some alternative binding proteins, such as miniproteins and DARPins, exhibit a resistance towards chemical denaturing or degradation by proteases. This renders them especially interesting for oral applications, as antibodies and antibody-derived fragments are degraded in the acidic gastrointestinal environment [36]. However, their efficient passage across epithelial barriers represents an unsolved problem.

Alternative binding proteins have been developed for various applications including therapy, diagnostics and imaging. Many of these scaffolds already reached late-stage clinical trials or have been FDA-approved, such as the miniprotein Ziconotide, once again demonstrating their immense potential [46]. One key aspect that must be considered with regard to therapeutic applications of these scaffolds is their immunogenic potential. However, previous studies have shown that even fully human antibodies can be immunogenic in humans [47], so a detailed evaluation of the immunogenicity of alternative scaffold proteins needs to be carried out independently [36]. Most scaffold proteins currently in clinical trials, however, are either derived from human proteins or comprise a low immunogenic profile [36]. Other scaffolds, such as affibodies, are mostly evaluated for short-lived applications, i.e. imaging rather than for therapeutic purposes.

**Fig. 2:**
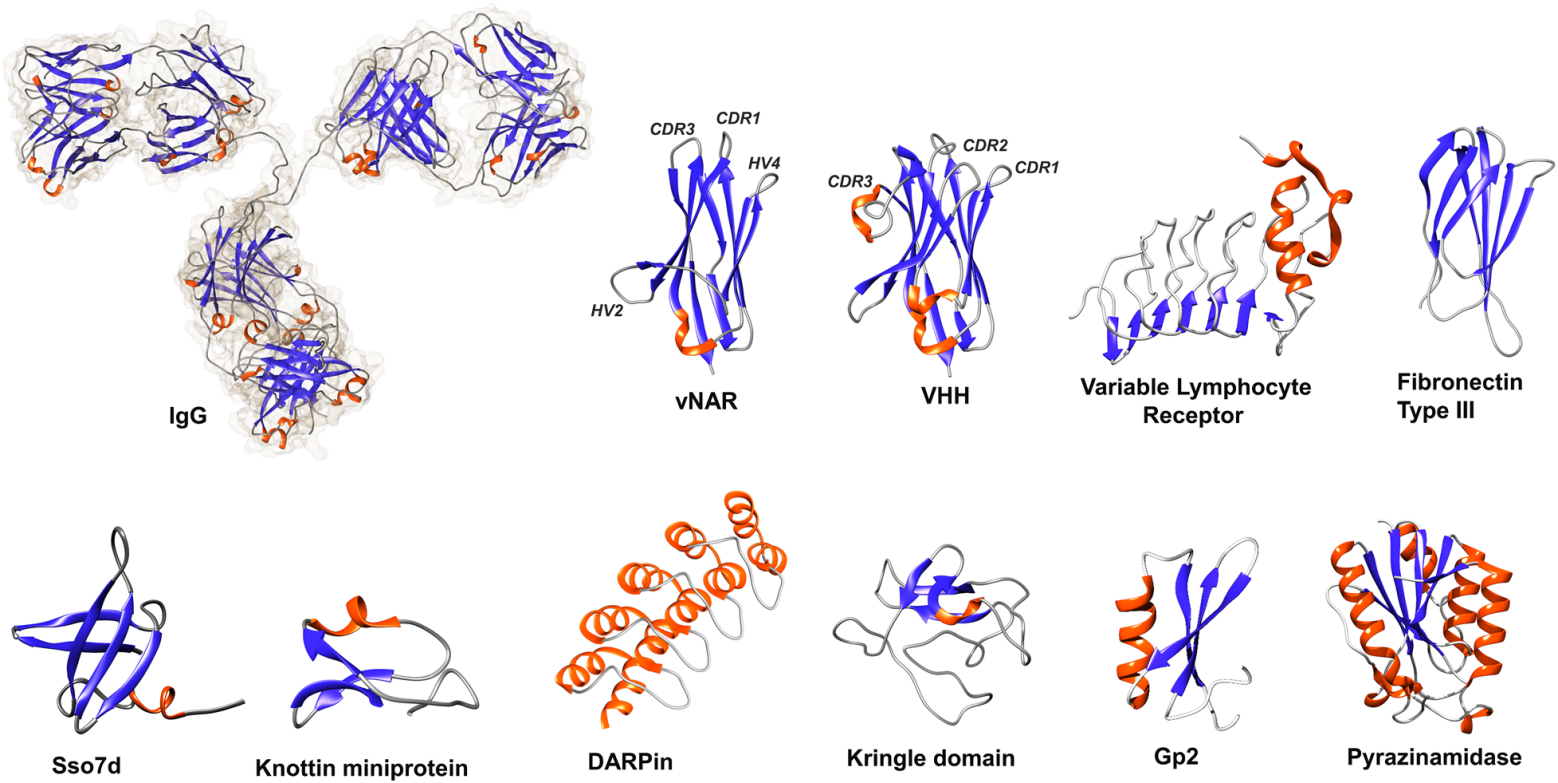
Structural depictions of the alternative scaffold proteins discussed in the scope of this review. Structures were prepared from pdb identifiers 1IGT (IgG), 4GHK (vNAR), 5HDO (VHH), 3G3B (Variable Lymphocyte Receptor), ITTG (fibronectin domain III), 1SSO (Sso7d), 1HA9 (oMCoTI-II; knottin), 2XEE (DARPin), 1HPK (kringle domain), 2WNM (Gp2) and 1ILW (pyrazinamidase) using UCSF Chimera [133]. The proportions of the depicted scaffold proteins are relative and do not reflect the actual differences in size. Secondary structures are colored in red (helices) and blue (β-strands). Distinct hypervariable loops discussed in the scope of vNAR and VHH scaffolds are annotated

Alternative scaffold proteins have been obtained and engineered using various display techniques and strategies for isolating variants with tailor-made properties. Specific examples that employed yeast display as high throughput platform are detailed below (Fig. 2). For some scaffolds, the eukaryotic expression machinery of yeast cells can be especially advantageous due to the presence of a high number of disulfide bonds as it is the case for miniproteins or Ig-derived scaffolds. Table 1 gives a detailed overview over the literature that is discussed in this review article with regard to the different scaffolds and enzymes that have been engineered using YSD.

**Table 1:** Published alternative scaffold proteins and enzymes which have been engineered using yeast surface display

Engineered scaffold or enzyme	References
Fn3 domain of fibronectin	Lipovsek et al. [24] Koide et al. [52] Hackel et al. [51] Koide et al. [40] Hackel et al. [55] Mann et al. [57] Sha et al. [58] Heinzelman et al. [59] Park et al. [49]
Human kringle domains	Lee et al. [25] Lee et al. [66]
DARPins	Schütz et al. [70]
Cystine-knot miniproteins	Silverman et al. [74] Silverman et al. [75] Kimura et al. [76] Lahti et al. [77] Kimura et al. [78] Glotzbach et al. [79] Maaß et al. [80]
Sso7d	Gera et al. [84] Gera et al. [85] Traxlmayr et al. [86] Kauke et al. [87]
T7 phage gene 2 protein (Gp2)	Kruziki et al. [89] Chan et al. [90]
Pyrazinamidase	Strauch et al. [91]
vNAR domains	Zielonka et al. [26] Zielonka et al. [95] Könning et al. [27] Könning et al. [96]
VHH domains	Ryckärd et al. [110]
Variable lymphocyte receptor (VLR)	Tasumi et al. [14] Hong et al. [114]
Horseradish peroxidase	Lipovsek et al. [122] Antipov et al. [123]
Sortase A	Chen et al. [124]
Lipases	Han et al. [125] White and Zegelbone [126]
β-lactamase	Cohen-Khait and Schreiber [127] Cohen-Khait et al. [128]
Glucose oxidase	Ostafe et al. [129]
*Nonribosomal Peptide Synthetase Adenylation Domain*	Zhang et al. [130]
Tobacco Etch Virus protease	Yi et al. [131]

### Engineering alternative binding proteins with yeast surface display

#### Fibronectins

Engineering of the 10th type III domain of fibronectin (termed Fn3 hereafter) in terms of its use as a novel scaffold protein was first described by Koide and coworkers in [48]. Fibronectins belong to the immunoglobulin superfamily (IgSF) and consist of several repeats of overall three different domains, namely FNI, FNII and FNIII [48]. In contrast to other proteins belonging to the IgSF, the Fn3 domain does not comprise any disulfide bonds. Its monomeric structure is composed of seven ß-strands, resembling conventional VH domains with three solvent-exposed loops that mediate binding [49]. Moreover, this monomeric architecture as well as the absence of disulfide bonds allows for a facile expression of Fn3 domains in *E. coli* cells [50].

Koide and coworkers were the first to describe the engineering of Fn3 domains for the purpose of molecular recognition. They elegantly demonstrated that highly specific Fn3 binders targeting ubiquitin could be isolated from a phage-displayed library that was comprised of Fn3 domains with randomized amino acids in two surface-exposed loops. They further characterized the structural integrity of a dominant Fn3 single clone, showing that this variant tolerated 12 mutations out of 94 residues and emphasizing the potential of Fn3 as alternative binding scaffold [48]. Although their approach involved phage display as the platform technology, it was later shown by Lipovsek and coworkers that Fn3 domains are also compatible with yeast display [24]. Their engineering approach focused on the generation of several Fn3 yeast libraries with mutations in either one or two loops of the protein scaffold. Both libraries were sampled towards hen egg lysozyme and subsequent affinity maturation of initial binders upon loop shuffling and recursive mutagenesis yielded variants with picomolar affinities. In a follow-up investigation, Hackel and colleagues further improved the affinity maturation process of yeast-displayed Fn3 domains yielding binders against lysozyme with single-digit picomolar affinities [51].

Using yeast surface display, Koide and coworkers demonstrated the feasibility of a binary code interface comprising serine and tyrosine residues for the diversification of Fn3 domains [52]. Their work illustrates that this minimal amino acid diversification approach is a valid strategy not only for obtaining high-affinity Fab fragments (as demonstrated previously [53]) but also for acquiring smaller alternative scaffold proteins. The success of this approach seems to stem from the ability of tyrosine residues to form a plethora of different nonbonded interactions as well as from the remarkable conformational diversity of the Fn3 loops that extent the rather limited chemical diversity.

The generation of mutant Fn3 libraries in the yeast as well as phage display format has been achieved not only by randomizing loop residues, but also upon the diversification of amino acid residues present in the protein backbone. Using both phage and yeast display, the feasibility of using a “side-and-loop” library was demonstrated [40]. This approach was based on mutations in the conventional loop regions but also extended to the ß-sheet surfaces [40], since previously generated Fn3/antigen co-crystal structures revealed a convex binding surface formed by one of the loops together with the face of a β-sheet [54]. The employment of the “side-and-loop” library led to the isolation of several high-affinity Fn3 domains towards overall three different target antigens, giving important insights into the design of novel molecular recognition surfaces.

In 2012, Hackel and coworkers isolated high-affinity fibronectin domains targeting various epitopes of the epidermal growth factor receptor (EGFR) using yeast surface display [55]. The identified Fn3 domains efficiently downregulated EGFR on multiple EGFR-overexpressing cell lines when reformatted as heterodimers. Chen and coworkers developed an extensive protocol for the isolation of Fn3 domains from yeast-displayed libraries [56]. Mann and colleagues used Fn3 domains in combination with yeast surface display for the identification of binders specifically targeting a distinct surface patch of the mitogen activated protein kinase (MAPK) Erk-2 [57]. They applied screening procedures including positive and negative selection steps. Positive selection steps relied on wildtype Erk-2 while negative selections encompassed a mutant version of Erk-2, leading to the enrichment of Fn3 domains specifically targeting the desired patch on the kinase surface. In another investigation, Sha and coworkers utilized yeast display screenings for the isolation of Fn3 domains towards the *N*- and *C*-terminal SH2 domains of the Src-homology 2 domain-containing phosophatase 2 (SHP2), a subunit of the multiprotein complex of the therapeutically relevant tyrosine kinase BCR-ABL [58]. Initial libraries were screened using phage display while additional mutagenesis and subsequent translation into the yeast display format yielded the final candidates. Using YSD, Fn3 domains were also engineered towards pH-sensitive target binding. To this end, Heinzelman and colleagues aimed for a significant decrease in antigen affinity at an endosomal pH of 5.5 [59]. Such pH-sensitive Fn3 domains might be useful for continuous receptor down-regulation in a therapeutic manner, allowing the release of the fibronectin from its receptor target in the acidic endosome [60]. Heinzelman and coworkers chose a site-directed mutagenesis approach that focused on the mutation of distinct framework rather than loop residues to histidine. The mutated positions were determined using a structure-guided algorithm. The resulting variants were analyzed on the yeast surface in terms of pH-sensitive binding to their antigen EGFR and yielded several Fn3 domains with the desired characteristics. Recently, Park et al. engineered yeast-displayed Fn3 domains towards binding of the tumor biomarkers ephrin type-A receptor 2 (EphA2) [49]. Selected single clones bound human EphA2 with single-digit nanomolar affinities and one candidate was shown to function as an in vivo imaging probe in mouse xenograft models.

Conclusively, yeast surface display of fibronectin scaffolds has yielded an array of binders towards various therapeutically relevant targets, often with impressive affinities for their respective antigens. The approaches described in this section further underline the versatility of this scaffold as well as its therapeutic relevance [37, 50]. However, it should be noted that an impressing variety of high-affinity Fn3 domains has also been generated upon employing phage [48, 61] and mRNA display [62]. As Fn3 domains are devoid of any disulfide bonds or glycosylation sites, they are compatible with bacterial surface display formats. Moreover, it has been demonstrated that the combination of phage and yeast display for the isolation of Fn3 domains is a feasible approach that harnesses the advantages of both display technologies [40, 52].

#### Human kringle domains

Kringle domains are ubiquitous domains which are present in all eukaryotic organisms [25, 63, 64]. These modular structural domains are composed of 78–80 amino acids and are present in many functionally distinct proteins. Most kringle domains can be found in the blood plasma in the form of coagulation factors or growth factors. As such, they are believed to mediate binding interactions with various proteins, lipids and small molecules. Structurally, kringle domains have a rigid core structure that contains three disulfide bonds and two β-sheets forming a triple-loop architecture [65]. Although kringle domains have evolved to participate in various biological functions, their framework regions display a high degree of sequence homology and sequence diversity is mainly found in the loop regions. Lee and colleagues engineered a novel binding scaffold based on human kringle domains by mutating 45 residues in the divergent loop regions [25]. Towards this end, they created a yeast-displayed kringle domain library based on kringle domain 2 from human plasminogen (Png) and screened towards the anticancer targets death receptor 4 and 5 (DR4; DR5) as well as tumor necrosis factor α (TNFα) [25]. The yeast library was created through synthetic shuffling and upon introducing randomized DNA codons. These codon combinations were selected with the aim of introducing preferably hydrophilic amino acids, thereby mimicking naturally occurring kringle domain sequences. After two rounds of magnetic sorting and three rounds of selection using FACS, several clones for each target were obtained and recombinantly expressed in the yeast *Pichia pastoris*. The correct connectivity of the disulfide bonds was verified using mass spectrometric analysis. The affinities of the expressed clones ranged from micromolar to two-digit nanomolar values. The recombinantly produced kringle domains were evaluated regarding their thermal stability and it was observed that, although subjected to extensive mutagenesis of 45 residues, their overall stability was not severely affected in comparison to the wild-type kringle domain that served as starting material for library generation. Subsequent experiments focusing on the biological activity of the isolated constructs yielded four variants targeting DR4 or DR5 that were able to induce dose-dependent cell death of various cancer cell lines. Out of the identified TNFα-targeting kringle domains, only one neutralized TNFα-mediated cytotoxicity in a concentration-dependent manner [25].

The results obtained by Lee and coworkers introduce a novel protein to the repertoire of alternative binding scaffolds which can successfully be combined with yeast display. In follow-up work, bispecific and bivalent kringle domains have been generated upon loop grafting of individual candidates and upon employing the yeast surface display technology [66]. As human kringle domains comprise a conserved pattern of three disulfide bonds, the utilization of yeast and, therefore, its eukaryotic expression machinery is potentially highly advantageous as it aids the correct folding of these complex molecules. The presence of chaperones and foldases in yeast provides an excellent environment for the generation and surface display of correctly folded proteins, increasing the chance of isolating functional binders and avoiding the presentation of misfolded and potentially “sticky” protein variants.

#### Designed ankyrin repeat proteins (DARPins)

Designed ankyrin repeat proteins are non-immunoglobulin proteins that consist of multiple, usually 33 amino acid long repeats [38, 67]. Structurally, each of these repeats comprises a β-turn that is followed by two α-helices. Although individual lengths of up to 29 consecutive repeats can be found, a number of as much as 4–6 repeats is commonly observed. Plückthun and coworkers pioneered the design of DARPin libraries that comprise *N*- and *C*-capped self-compatible repeats, generating scaffolds with high stability [31]. Through structural analyses, they identified and chose for mutagenesis conserved framework and surface residues that were more diverse among many naturally occurring repeats. In that manner, they generated both ribosomal and phage libraries with great success [68, 69]. A recent publication from Schütz and colleagues focused on the isolation of ankyrin repeat protein scaffolds which specifically interact with malachite green, thereby activating the fluorophore [70]. Such fluorogen-activated proteins (FAPs) are useful tools for various biological applications, such as imaging. Although many FAPs have been reported to date, all of these proteins are single-chain variable fragments (scFv) that specifically interact with the desired dye. As the application of scFvs as FAPs has some limitations, such as their relatively low stability and the requirement of intramolecular disulfide bonds for accurate folding, FAPs based on scaffold proteins represent an attractive alternative. In their investigation, Schütz and coworkers utilized ribosomal display and subsequent selections using yeast surface display for an ankyrin repeat protein domain that recognizes malachite green and functions as a FAP. Two different ribosomal display libraries comprising either two or three internal protein repeats were screened. However, binders to malachite green were only enriched from the three-repeat library and further subjected to affinity maturation using mutagenic PCR and subsequent screenings in a yeast display format. After three rounds of mutagenesis and two rounds of screening in each case, the affinity-matured pool of binders exhibited significantly enhanced fluorescence signals after the addition of malachite green. Interestingly, the best performing binder that was used for further biochemical characterizations and crystallization experiments revealed the formation of a homodimer upon binding to the dye. Further experiments using the identified DARPin unambiguously demonstrated its potential to function as a selective labeling tool for proteins on the cell surface as well as in the cytosol [70].

This work nicely demonstrates the combination of ribosomal as well as yeast display for the isolation of DARPins that can act as FAPs and points out the synergy that is achieved upon combining these two powerful display methodologies. In contrast to ribosomal display, yeast display allows functional screening for enhanced fluorogenic activation of malachite green upon using fluorescence-activated cell sorting.

#### Cystine-knot miniproteins

Cystine-knot miniproteins, also referred to as knottins, are a class of naturally occurring cystine-rich peptides [71, 72]. They are characterized by their defined three-dimensional and rigid structure which is attributed to the presence of structurally constraining disulfide bonds. Two disulfide bridges form a macrocycle which is penetrated by the third bond, forming an interlocked arrangement. Owing to this structural feature, cystine-knot miniproteins are highly stable towards proteolytic and chemical degradation and also exhibit increased thermal robustness [34]. Miniproteins have been isolated from a variety of species, including vertebrata, arthropoda, fungi, plantae, porifera and mollusca, indicating evolutionary convergence. In several rational engineering approaches, it has been shown that these scaffolds can tolerate a variety of mutations, culminating in the removal of their actual binding loop and grafting of other loop structures addressing different target proteins [72, 73]. Due to their small size of about 30–50 amino acids, recombinant as well as chemical synthesis can be employed, rendering them attractive as scaffold-based affinity reagents.

In 2009, Silverman et al. were the first to report the successful employment of yeast surface display for the isolation of integrin-binding agouti-related protein (AgRP) variants targeting α_v_β_3_ integrins [74]. The AgRP variant utilized for their approach contained overall four disulfide bonds which formed four solvent-exposed loop structures capable of binding to a given protein of interest. Silverman and colleagues decided to replace loop number 4, the most solvent-exposed binding site, with a 9-amino acid RGD-motif loop from the 10th domain of fibronectin. This RGD-motif has been reported to be essential for integrin binding. Subsequently, they randomized the RGD flanking sequences and generated a library that was displayed on the surface of yeast. The miniprotein variants which were obtained after seven rounds of FACS selection exhibited antibody-like affinities as well as high specificity for α_v_β_3_ integrins as determined in cellular assays using both recombinantly expressed and chemically synthesized knottins. Recombinant expression of the isolated AgRP proteins was accomplished in the yeast *P. pastoris*, affording the benefit of an accurate folding machinery for these structurally demanding proteins. Silverman and colleagues conducted additional experiments encompassing the isolation of AgRP variants targeting different integrins [75]. In this study, a six-amino acid containing loop of AgRP was replaced with a nine-amino acid motif containing the RGD sequence as well as randomized flanking sequences. Using yeast surface display, miniprotein variants specifically engaging the α_IIb_β_3_ integrin or both, α_IIb_β_3_ and α_v_β_3_ were identified. These miniproteins exhibited high efficacy in platelet aggregation inhibition assays and might bear potential as thrombosis inhibitors.

Similarly, Kimura et al. combined this extraordinary class of scaffolds with yeast surface display for the isolation of EETI-II variants targeting two different integrin types [76]. The framework for their engineering approach was a miniprotein isolated from the squirting cucumber *Ecballium elaterium*, EETI-II, a member of the squash family of trypsin inhibitors. Kimura and coworkers replaced the 6-amino acid, trypsin-binding loop with an 11-amino acid, integrin-binding sequence from a fibronectin domain that already comprised the RGD peptide motif. Subsequently, the flanking amino acids were randomized and the resulting yeast library was screened towards α_v_β_3_ and α_v_β_5_ and yielded binders in the double-digit nanomolar range. Moreover, Lahti et al. utilized yeast surface display in order to thoroughly investigate the EETI-II knottin scaffold with regard to tolerated loop sequence diversity and loop lengths [77]. Their findings allow a distinct prediction of admissible amino acid residues at certain positions and detailed predictions regarding loop lengths, which is crucial for accurate folding and hence biological activity of miniproteins. Besides mutating solely one of the three loops in EETI-II, follow-up work published by Kimura and colleagues in 2011 demonstrated that diversification of two adjacent loops can likewise result in the isolation of high-affinity miniproteins targeting different integrins [78]. Actual loop sizes were expanded from 6 and 5 amino acids natively found in the loops to 11 and 10 residues in the engineered counterparts.

Knottin variants based on the AgRP and EETI-II scaffold are not the only variants successfully displayed and characterized using yeast surface display. In 2013, Glotzbach and colleagues were able to utilize open-chain derivatives of the trypsin-inhibitors MCoTI-II (*Momocordia cochinchinensis* trypsin inhibitor) and SOTI-III (*Spinacia oleracea* trypsin inhibitor) for the generation of combinatorial libraries in yeast [79]. Based on structural elucidations previously performed on cystine-knot miniprotein scaffolds, only distinct amino acid residues were allowed at certain loop positions in the MCoTI-II variants displayed on yeast. Randomization of the 10-amino acid, trypsin-binding loop of SOTI-III was carried out upon employing codon-degenerated mutagenesis. Both libraries were screened towards matriptase-1, a transmembrane serine protease that is involved in tumor metastasis, and yielded overall three MCoTI-II and one SOTI-III based single clones. These variants were subsequently synthesized using solid-phase peptide synthesis (SPPS) and oxidative folding was carried out in order to ensure correct disulfide connectivity. Bioactivity assays of each isolated knottin confirmed the correct folding of chemically synthesized miniproteins and further demonstrated the feasibility of the described, yeast display-based approach. In another attempt recently conducted by Maaß and colleagues, randomized miniprotein variants based on MCoTI-II were screened against the immune checkpoint receptor cytotoxic T lymphocyte‐associated antigen 4 (CTLA-4) using yeast surface display [80]. The most potent variant was chemically synthesized and subjected to a wide array of oligomerization approaches that relied on the conjugation to a human IgG_1_ Fc, the C4 binding protein and neutravidin in order to increase affinity for CTLA-4. Maaß and coworkers could show that it is possible to create avidity effects upon oligomerization of the miniprotein scaffold, thereby decreasing the dissociation constant from micromolar to single-digit nanomolar values [80].

While knottin library screening using phage display has been applied successfully, yeast display is particularly advantageous for several reasons. When using phage display, special attention has to be paid to potential knottin disulfide scrambling with cysteines of the pIII display protein. Moreover, as mentioned above for the cystine-rich kringle domains, misfolded miniprotein variants are thought to be degraded by the yeast unfolded protein response machinery and, therefore, excluded from surface display.

#### The Sso7d protein

In the past years, an additional protein scaffold which is characterized by its extraordinary thermal stability has attracted the attention of protein engineers. The DNA-binding protein Sso7d is derived from the hyperthermophilic archaeon *Sulfolobus solfataricus* and exhibits an Src SH3-like three-dimensional structure [81, 82]. Its amino acid sequence lacks cysteine residues and glycosylation sites and tolerates extensive mutagenesis without largely compromising the protein’s melting temperature of about 100 °C [83]. As has been demonstrated by Gera and colleagues in 2011, the Sso7d scaffold comprises not only high thermal stability but also robustness towards extremely high and low pH values as well as denaturing agents [84]. More importantly, it was demonstrated that this unique scaffold could successfully be combined with yeast surface display in order to isolate highly specific binders addressing fluorescein, a peptide fragment form β-catenin, hen egg lysozyme, streptavidin and several immunoglobulin isotypes. Selected binders were recombinantly expressed in the *E. coli* cytoplasm in good yield, offering an additional advantage over conventional antibodies which are typically expressed in mammalian cells. In another approach, Gera and coworkers engineered Fc-targeting Sso7d variants towards pH-sensitive target binding [85]. Therefore, two different techniques were employed: histidine scanning of a distinct Fc-binding Sso7d variant as well as random mutagenesis of a population of Fc-binding candidates. The latter approach yielded a sublibrary which was subjected to several rounds of fluorescence-activated cell sorting using alternating selections at neutral and acidic pH values. Both strategies led to the identification of several pH-sensitive Sso7d variants with affinities in the nanomolar range. All isolated variants exhibited significantly reduced binding to the Fc domain at pH 4.5.

Since the Sso7d scaffold is a DNA-binding protein with a very positive net charge, it is potentially difficult to prevent non-specific interactions of this protein with anionic interfaces, i.e. the surface of mammalian cells [86]. Additionally, the actual binding interface of Sso7d is surrounded by a ring of positively charged lysine residues that can potentially impair interactions with positively charged target proteins. In order to circumvent this potential obstacle, Traxlmayr and coworkers engineered a reduced-charge Sso7d scaffold that comprised a decreased number of lysine residues, yielding several variants with neutral net charge [86]. Importantly, the reduced-charge variants almost completely retained their extraordinary thermal stability. The most stable scaffold was chosen as starting point for the generation of two Sso7d yeast libraries that allowed either 11 or 18 amino acids at overall 9 solvent-exposed positions in the protein’s binding interface. Traxlmayr et al. showed that both libraries yielded binders towards several differentially charged epitopes on EGFR as well as mouse serum albumin. The identified binders were produced in *E. coli* cells in good yield and maintained their extraordinary thermostability while exhibiting nanomolar affinities. Their approach further validates the compatibility of this highly stable alternative scaffold protein with yeast surface display and demonstrates its robustness and tolerance towards structural modifications. In a more recent investigation, Kauke et al. utilized the same libraries for the isolation of Sso7d mutants that revealed a preferential binding for the G12D mutant form of the GTPase K-ras over the wildtype enzyme [87]. Mutant K-ras is one of the major factors driving oncogenesis and tumor progression in a variety of solid tumors and represents an attractive target for cancer research. Using their highly specific Sso7d binder, Kauke and coworkers were able to generate co-crystal structures of the mutant as well as wildtype forms of K-ras in complex with Sso7d. These structures provide valuable insights into the state of switch I that is essential for K-ras signaling. The isolation of a mutant-specific K-ras binder offers a platform for the design of future Ras inhibitors and represents a novel tool for the continuing exploration of Ras biology.

#### T7 phage gene 2 protein (Gp2)

Using the Protein Data Bank, Kruziki and coworkers identified the T7 phage gene 2 protein (Gp2) as a suitable protein scaffold for the engineering of molecular recognition [41]. Their approach relied on the screening of the available protein structures with regard to defined structural criteria which define an ideal protein scaffold. The criteria were, among others, derived from the topology of antibody as well as fibronectin domains, which comprise diversifiable loops at the end of β-sandwiches. In addition, other important aspects such as a small size, β-sheet content, absence of disulfide bonds, the number of solvent-exposed loops as well as the solvent-accessible surfaces were taken into consideration. The T7 phage gene 2 protein (Gp2) was subsequently identified as the ideal protein structure. Gp2 is a 67-amino acid long *E. coli* RNA-polymerase inhibitor derived from the T7 phage [88]. Kruziki et al. further minimized the Gp2 protein in order to generate a 45-amino acid scaffold that was subsequently subjected to protein engineering using degenerate oligonucleotides for mutagenesis and yeast surface display as high-throughput screening system. Amino acids belonging to the two solvent-exposed loops were selected for mutation and randomized using oligonucleotide mixtures that encoded for an amino acid distribution mimicking antibody CDR regions. Upon screening of the initial Gp2 libraries towards the model antigens lysozyme, epidermal growth factor receptor (EGFR), rabbit and goat IgG, several rounds of affinity maturation were performed and Gp2 variants with affinities for their respective target in the low nano- to picomolar range were obtained. Recombinant expression of the Gp2 mutants was carried out in *E. coli* cells, whereas the expression of wild-type Gp2 was only detectable upon utilizing an *E. coli* strain that comprised a truncated form of its RNA-polymerase. This can most likely be attributed to Gp2’s native function as an inhibitor. All of the identified mutants comprised high thermal stability which, in some cases, even exceeded the melting temperatures obtained for the wild-type scaffold. In follow-up work by Kruziki and coworkers in 2016, the highly specific EGFR-targeting Gp2 variant was utilized as a molecular probe for PET imaging experiments in xenograft mouse model [89]. In a very recent investigation, Chan and coworkers could identify several Gp2 variants which specifically target the insulin receptor [90]. Upon randomizing two adjacent loops of the Gp2 scaffolds and mimicking the natural amino acid repertoire found in antibody variable regions as described above, several binders addressing the extracellular domain of the insulin receptor were isolated and affinity matured. The identified hit candidates exhibited low nanomolar affinities towards their target and, interestingly, contained two cysteine residues in their sequence which most likely enable the formation of a disulfide bond that is naturally absent in the wild-type protein. Importantly, all three Gp2 mutants showed insulin receptor-specific binding as well as inhibition in breast cancer cells.

The Gp2 protein represents a new addition to the scaffold proteins that can be engineered using yeast surface display. This scaffold has been identified upon employing a thorough data base research and upon considering the unique attributes of known alternative scaffold variants. As predicted, the Gp2 scaffold tolerated a variety of different mutations upon maintaining its excellent physicochemical robustness and yielded several biologically active variants.

#### Pyrazinamidase

In 2014, Strauch et al. engineered the enzyme pyrazinamidase from the hyperthermophile bacteria *Pyrococcus horikoshii* towards pH-dependent binding of the human Fc region [91]. Their investigation relied on the finding that most of the reported Fc-binding proteins, such as the relatively well-known Protein A, address the same “consensus” region located between the CH2 and CH3 domains. Using computational hotspot-guided protein interface design, they evaluated several bacterial scaffold proteins in terms of compatible binding sites. Their approach also considered histidine residues found in the Fc interface in order to design a pH-switchable protein scaffold. Several surface-engineered scaffolds were tested for binding in a yeast-display format. The most promising candidate was the one derived from the bacterial enzyme pyrazinamidase. This initial, re-designed protein variant was then further subjected to error-prone PCR in order to identify mutants that comprised a higher affinity at neutral pH, but decreased binding in an acidic environment. A yeast surface display library was constructed and screened for four rounds. The best performing scaffold variant was expressed in *E. coli* cells in good yield and showed remarkable physicochemical stability, even at temperatures of 80 °C. Proof-of-concept affinity purifications using the pH-sensitive scaffold as an affinity ligand yielded a pure IgG fraction upon employing milder elution conditions compared to standard Protein A purifications. This approach further emphasizes the potential of alternative scaffold proteins as biotechnological tools and represents another valuable contribution to the available scaffold repertoire.

#### The IgNAR variable domain (vNAR)

Cartilaginous fish possess a unique class of heavy-chain only antibodies termed immunoglobulin novel antigen receptor or IgNAR [92, 93]. These antibodies were first isolated by Flajnik and coworkers in 1995 from the serum of the nurse shark (*Ginglymostoma cirratum*) [94]. Antibodies of the IgNAR isotype comprise five constant domains and a single variable domain which is connected via a hinge-like linker region. The variable domains of IgNAR antibodies are termed vNAR domains and exhibit improved solubility due to the lack of a light chain (V_L_) interaction partner. The binding interface, which would normally mediate V_L_ chain pairing, therefore comprises an increased amount of hydrophilic amino acid residues at positions that usually exhibit hydrophobic moieties. VNAR domains additionally display extraordinary stability and are able to fold back into native conformation after heat-induced denaturation and subsequent cooling. With a size of approximately 13 kDa vNAR domains represent the smallest antibody-like antigen-binding entities known to date [92]. When taking a closer look at their structure it is noticeable that, in comparison to conventional as well as camelid antibodies, vNAR domains are lacking a CDR2 loop. Instead, they comprise unusually long CDR3 binding sites which, for the most part, are responsible for antigen binding. The much shorter CDR1 loop is likewise involved in mediating antibody-antigen interactions, although to a lesser extent. Besides CDR1 and CDR3, vNAR domains comprise two hypervariable loops (HV) termed HV2 and HV4. Diversification of the vNAR repertoire is mainly CDR3-based, however, randomization of the CDR1 binding site of a distinct vNAR domain has been found to be an excellent tool for affinity maturation [26]. The employment of yeast surface display as platform technology for the screening of vNAR libraries has resulted in the isolation of binders towards several disease-related target proteins, such as EpCAM, EphA2 and HTRA1 [26]. In this particular example, Zielonka and coworkers generated semi-synthetic vNAR yeast libraries upon using the antibody repertoire of non-immunized bamboo sharks (*Chylioscyllum plagiosum*) [26]. Subsequently, the CDR3 loops of these sequences were randomized upon employing a trimer-based oligonucleotide mixture, giving rise to a variety of different loop sequences while retaining the natural framework diversity found in this shark species. After initial screening rounds and the enrichment of vNAR domains targeting overall three different antigens with modest affinity, affinity-matured sublibraries were generated. Analogous to the initial trimer-based randomization of the CDR3 binding sites, the enriched vNAR domains were subjected to randomization of the CDR1 loop. Zielonka and coworkers demonstrated that incremental affinity maturation for all of the initially obtained binders was possible in this manner [26]. The improvement of affinity ranged from micromolar affinities to values in the single-digit nanomolar range after three rounds of screening. Recombinant expression of selected vNAR binders was accomplished upon reformatting them as maltose-binding-protein fusions and subsequent expression in *E. coli* cells.

Another approach recently investigated by Zielonka and colleagues encompassed the engineering of vNAR domains towards bispecificity using yeast surface display [95]. Towards this end, it could be shown that, despite their small size, these rigid protein scaffolds bear the potential of binding two different antigens simultaneously, making them the smallest bispecific molecular entities reported so far. In the scope of these investigations, trimer-based randomization of the HV2 loop was carried out. Based on previously identified vNAR crystal structures, HV2 rather than HV4 was chosen as it wraps around the bottom of the protein, rendering it an ideal starting point for the engineering of bispecificity. After the generation of a yeast library based on an EpCAM-binding vNAR domain, binders addressing EpCAM as well as CD3ε or the Fc domain of human IgG_1_ were obtained [95].

Recently, Könning et al. successfully employed high-throughput screening of semi-synthetic vNAR yeast libraries for the identification of pH-sensitive vNAR variants [27]. In this case, randomization of naïve vNAR scaffolds was accomplished through the employment of histidine-enriched and trimer-based oligonucleotide mixtures analogous to the library generation procedure as described by Zielonka et al. [26]. The rationale behind this approach was the establishment of a generic, semi-synthetic and histidine-enriched vNAR yeast library that allows for the selection of target-specific and pH-responsive vNAR domains in a single screening procedure. In contrast to previously published approaches that aimed at pH-engineering, this generic library omits the necessity of a two-step screening and engineering procedure. While the generation of a pH-responsive binder usually involves extensive histidine mutagenesis of an existing parental protein and, subsequently, the generation of a sublibrary that needs to be sampled, the approach published by Könning et al. allows for the de novo identification of vNAR domains which are antigen-specific and pH-switchable. In that manner, this process enables optimal sequence selection of target-binding and pH-responsive vNAR variants without the need for compromising the favorable properties of a parental binder which already comprises an optimal sequence prior to histidine-based mutagenesis. After the enrichment of antigen-binding vNAR domains during the first rounds of sorting, Könning et al. employed an alternating selection strategy that encompassed positive as well as negative selections at neutral or acidic pH, respectively. Reformatting of these pH-sensitive vNAR scaffolds was accomplished by fusing them to a human IgG_1_ Fc domain and expressing the fusion proteins in HEK293 cells. Since vNAR domains comprise a highly stable framework and a robustness towards a wide range of different pH values, salt conditions and temperatures, the authors propose that these pH-sensitive vNAR domains might serve as valuable affinity ligands for tailored chromatographic purification processes [27].

In a more recent approach, Könning et al. discovered that screening of the previously reported, histidine-enriched semi-synthetic vNAR libraries against therapeutic antibodies almost exclusively yielded anti-idiotypic binders [96]. This is particularly interesting as no counter selections were employed and the identified binders solely interacted with the variable rather than the constants domains of the antibody target. Even more astonishing was the finding, that the reformatted anti-idiotypic vNAR-Fc fusion constructs comprised affinities in the nano- to picomolar range, although derived from semi-synthetic and not immune libraries. This observation was unexpected considering that binders which were initially derived from such semi-synthetic libraries towards other antigens typically comprised bivalent affinities in the three-digit nanomolar to micromolar range [26, 27, 95].

Owing to their small size and increased stability in comparison to conventional antibodies and also scFv domains, vNAR domains represent an attractive protein scaffold that can easily be tailored towards function. It has been shown that this scaffold readily tolerates substitutions throughout its loop regions without compromising overall stability. The presence of a varying number of non-canonical disulfide bonds in these domains seems to predestine eukaryotic display systems for protein engineering. Yet, many approaches have clearly demonstrated the suitability of other display formats including phage and ribosomal display [97] for the screening of immune [98], naïve [99] and synthetic [100] vNAR libraries. Nevertheless, the application of yeast surface display as state-of-the-art screening tool allows for the rapid selection of high-affinity vNAR antibody domains, appending this scaffold to the growing list of alternative binders that can be engineered and sampled using this technology.

#### VHH domains

The identification of heavy-chain only antibodies in the serum of camelids was a serendipitous discovery made by Hamers-Casterman in 1995, expanding the repertoire of immunoglobulin subtypes [101]. Equally to shark-derived vNAR domains, the unique composition of camelid single domain antibodies affords the benefit of a paratope which is formed by only a single entity. This peculiarity is attributed to the presence of elongated CDR3 loops [102]. Due to their small size, VHH domains are able to address cryptic epitopes such as G-protein coupled receptors (CXCR4 and 7), which are difficult to address using monoclonal antibodies [103, 104]. Vincke and coworkers could show that, due to high sequence homology between VH and VHH domains, humanization of camelid VHH domains is a feasible method for the generation of variants which are potentially less immunogenic than the wildtype domain [105]. They were also able to demonstrate that these humanized variants are amendable to loop-grafting of other CDR structures, yielding a general platform for humanization. Although phage display still represents the standard technology for the isolation and identification of VHH domains (or “Nanobodies”) from immune [106], naïve [107] or synthetic libraries [108, 109], an attempt performed by Ryckärd and coworkers focused on the isolation of VHH domains using yeast surface display in combination with a glyco-engineered strain of the yeast *P. pastoris* [110]. Their library was based on genetic material obtained from blood lymphocytes of a llama that had been immunized with green fluorescent protein. The established VHH yeast library was genetically fused to the α-agglutinin protein from *S. cerevisiae*. After two rounds of sorting using FACS, a target-binding population was enriched. Overall, two single clones with affinities for GFP in the single-digit nanomolar range could be identified.

The experiments conducted by Ryckärd and coworkers unambiguously prove that yeast surface display can be combined with camelid-derived VHH domains in order to isolate high-affinity binders from an immune library. This approach represents an efficient addition to the standard phage display procedure commonly employed for the isolation of such antibody domains and could easily be expanded to naïve as well as synthetic VHH libraries.

#### Variable lymphocyte receptors (VLR)

Lampreys are members of the ancestral vertebrate taxon (jawless fish) which evolved a special immune repertoire of so-called variable lymphocyte receptors (VLRs) that consist of highly diverse leucine-rich repeats [111]. As such, VLRs represent the only known adaptive immune system that is not based on immunoglobulins. In 2009, Pancer and coworkers reported the isolation of monoclonal VLRs from large VLR libraries that were derived from antigen-stimulated and also from naïve animals [14, 112]. To this end, a yeast surface-display vector was constructed which fused the VLRs *C*-terminal to the yeast Flo1p surface anchor. The yeast flocculation protein Flo1p has a stalk-like structure and a *C*-terminal GPI cell surface anchor motif that can be used to display recombinant proteins on the surface of yeast [113]. Although Flo1p cell surface density was found to be one order of magnitude lower compared to the Aga1p/Aga2p display anchor, binders with single-digit picomolar affinities targeting several enzymes (lysozyme, β-galactosidase, cholera toxin subunit B) and other proteins could be obtained. Moreover, error-prone PCR was used to introduce substitutions along the diversity region of a weak binding anti-lysozyme VLR. The resulting mutant library was subjected to FACS screenings, resulting in several unique clones with 100-fold improved affinity for lysozyme compared to the wild-type VLR.

Interestingly, Hong et al. described a strategy for YSD selection of glycan-binding VLRs that revealed selective and high affinity binding to glycans and glycoproteins. One VLR was used to detect cancer-associated carbohydrate antigens in 14 different types of cancers in human tissue microarrays [114]. Hence, it can be concluded that VLRs may be useful natural single-chain alternatives to conventional antibodies for a broad range of therapeutic and biotechnological applications.

Although the YSD approaches described in this section do not encompass the classical Aga2p system, they demonstrate the feasibility of combining this screening platform with VLR antibodies. The isolation of high-affinity VLR domains from naïve and immune repertoires could be accomplished upon utilizing an alternative cell wall anchor protein that resulted in the presentation of a lower copy number of VLR fusion proteins in contrast to the Aga1p/Aga2p system. In addition to using YSD, it has also been shown that modified VLR domains (dVLR) are compatible with phage display [115, 116]. Although the phage display campaigns yielded several VLR-based binders, the efficient recombinant production of the engineered VLR scaffolds in bacterial hosts represented a drawback [115]. Lee and colleagues addressed this problem upon redesigning the N-terminal VLR region and generating a VLR-based scaffold termed “Repebody”. Binders which were selected from phage libraries comprising diversified Repebodies could be produced in *E. coli* cells in good yield. Taken together, these results indicate that the utilization of a eukaryotic expression machinery [117] could potentially be superior to bacterial expression and display systems when it comes to native VLR antibodies and engineered VLR-based scaffolds.

## Enzyme engineering using yeast surface display

Protein engineering of enzymes using directed evolution has become a valuable tool to improve reaction kinetics, increase stability or alter the substrate specificity of the desired enzyme [10, 118, 119]. Utilizing yeast display for the engineering of enzyme variants has gained increasing attention over the past years, as yeast cells offer an adequate posttranslational modification machinery suitable for the expression of more demanding proteins. Conventional enzyme screening approaches usually involve colony-screening or microtiter plate assays, as it is crucial to employ a screening system that enables an efficient genotype–phenotype linkage [10, 120, 121]. In the following section, we will focus on screenings that include yeast surface display together with fluorescence-activated cell sorting for the identification of enzyme candidates with superior functionality. As not only the improvement of binding can be a crucial parameter for certain proteins, we think it is of major importance to emphasize functional screenings that have been conducted using yeast display. The following examples underline the versatility of this platform for addressing a variety of different criteria other than affinity.

### Horseradish peroxidase

In 2007, Lipovsek and colleagues engineered horseradish peroxidase (HRP) towards enhanced enantioselectivity upon employing yeast surface display [122, 123]. Their approach involved two different randomization strategies: One library was constructed upon performing error-prone PCR on the complete HRP gene, whereas the second library solely sampled five specific residues which were located close to the active site of the enzyme. Subsequently, a screening procedure for the identification of enantioselective variants was established, including several positive as well as negative selection steps for the D- or L-enantiomer, respectively. Interestingly, only the active-site mutant library yielded binders with the desired specificity.

### Sortase A

Another enzyme engineering attempt involving yeast surface display was conducted by Chen and coworkers who identified mutant variants of the *Staphylococcus aureus* enzyme Sortase A with improved catalytic activity [124]. Sortase A specifically recognizes proteins comprising an LPXTG amino acid motif and subsequently connects them covalently with GGG-containing counterparts. The yeast display setup developed by Chen and colleagues involved the fusion of mutated Sortase A variants to Aga2p, while Aga1p was equipped with a reactive handle. This handle is used in order to enzymatically link an LPETG-functionalized substrate to the cells. Upon adding a second substrate comprising an *N*-terminal GGG motif as well as an affinity handle such as biotin, active Sortase variants were able to link the two substrates together upon forming a covalent linkage. Successful attachment of the GGG-containing substrate could be verified upon immunofluorescence stainings that rely on fluorescently-labeled streptavidin reagents.

### Lipases

In 2011, Han and colleagues isolated *Rhizomucor miehei* lipase variants with improved esterification activity in organic solvents [125]. Their yeast-surface display approach relied on the yeast *P. pastoris* and an *N*-terminal Flo1p anchor that tethered the mutated lipase variants to the cell surface. A combination of multiple sequence alignments and site-directed mutagenesis was applied in order to identify lipase mutants with prescribed catalytic activity. In another approach conducted by White and Zegelbone, yeast surface display was utilized for the improvement of catalytic activity. Their research focused on the *E. coli* lipoic acid ligase [126]. The yeast display screenings relied on the constitutive expression of a reactive handle and inducible expression of mutated ligase variants. Overall, four rounds of screening were necessary in order to achieve the desired ligation activity.

### β-lactamase

Yeast surface display was also used to investigate the plasticity of the interface of TEM1 β-lactamase with its protein inhibitor BLIP by low-stringency selection of a random enzyme library [127]. To this end, Cohen-Khait and Schreiber generated an error-prone PCR library of 10^8^ clones which underwent selection against binding to fluorescently labeled BLIP by fluorescence-activated cell sorting. The gene composition of the resultant variants was subsequently evaluated by deep sequencing. The authors could show that most interfacial residues could be mutated without a loss in binding affinity, protein stability, or enzymatic activity, suggesting high plasticity in the interface composition. By drastically decreasing the library—ligand incubation time to 30 s, Cohen-Khait and Schreiber were able to specifically select for faster-associating protein complexes, a methodology that may also be useful for the generation of tightly binding enzyme inhibitors from combinatorial libraries. In follow-up work, the Schreiber group utilized yeast surface display for the engineering of a self-interacting TEM1 β-lactamase that is able to form homodimeric complexes, a feature that is uncommon for this particular type of β-lactamase [128]. They comprehensively demonstrate that mutation of solely two to four amino acids in a pre-stabilized TEM1 scaffold is sufficient to enable binding to wildtype TEM1. However, transferring these mutations from the pre-stabilized protein to the wildtype TEM1 scaffold and transforming *E. coli* cells with the respective plasmid resulted in the expression of a non-functional enzyme that was not able to confer ampicillin resistance to the cells. Their work sheds light on so-called “structural gatekeeper” mutations which can, theoretically, introduce de novo binding sites to existing proteins, but due to structural instability of the resulting mutant protein do not evolve. Thereby, this mechanism avoids the formation of unwanted interaction sites and unfolding of the respective protein.

### Glucose oxidase

The enzyme glucose oxidase (GOx) is used in many industrial processes. Ostafe and coworkers described a sophisticated procedure to isolate GOx variants with fivefold enhanced activity from a library of 10^5^ variants upon employing yeast surface display [129]. Yeast cells expressing GOx enzyme variants were encapsulated in water-in-oil single emulsions together with the components needed for the enzymatic reaction. Active GOx variants subsequently started an enzymatic cascade that led to the staining of the surface of yeast cells with tyramide-fluorescein. After breaking the emulsion, yeast cells were further stained with antibodies and analyzed by FACS. This method enabled the quantitative screening of GOx libraries with the aim to identify clones with improved specific activity.

### Nonribosomal peptide synthetase adenylation domain

In an elegant approach published by Zhang and colleagues in 2013, the yeast surface display system was employed for engineering the adenylation domain of nonribosomal peptide synthetase, an enzyme that produces natural product molecules of complex structures such as penicillin, vancomycin, and daptomycin [130]. The authors took advantage of the high-affinity binding of a substrate-adenosine monosulfomate (AMS) derivate. Screening of a library using biotinylated AMS probes allowed for the isolation of active enzymes directly on cells upon labelling with a streptavidin-fluorophore conjugate followed by FACS selection. This strategy was successfully used to engineer the substrate specificity of DhbE, an adenylation domain that activates 2,3-dihydroxybenzoic acid for the synthesis of the natural product bacillibactin. DhbE mutants were identified that preferably recognize non-native substrates such as 3-hydroxybenzoic acid for the adenylation reaction.

### Tobacco Etch Virus protease

Yi and colleagues utilized yeast surface display in combination with FACS for the isolation of *Tobacco Etch Virus* proteases with improved sequence specificity and accelerated proteolytic cleavage [131]. Their display approach encompassed the co-expression of a mutant protease with an endoplasmatic reticulum (ER) retention sequence as well as an Aga2p fusion of several protease substrates and counterselection sequences. Both, the substrate fusion protein and the protease mutant are under the control of a bidirectional, galactose-inducible promotor. An ER retention sequence at the end of the protease and the sequence fusion protein results in close proximity of the constructs at the ER and subsequent cleavage of either the substrate or the counterselection sequences by the protease variants. Upon cleavage, the ER retention signal is removed and the resulting substrate construct is secreted and tethered to the yeast surface via the Aga2p anchor. Different epitope tags which are fused in between the counterselection and the correct substrate sequence allow for specific detection of desired protease mutants. In this manner, Yi and colleagues were able to isolate TEV protease variants specifically recognizing an altered sequence motif [131].

## Conclusion

Alternative scaffold proteins have emerged as powerful tools for specific molecular recognition with regard to applications in therapy, diagnostics and biotechnology. As such, they have demonstrated their potential as alternative tools to antibodies which are usually the common choice when specific protein binders are required. Many advances have been made in the field, with several alternative scaffold proteins currently being investigated in clinical trials. For the purpose of generating highly specific scaffolds, directed evolution in combination with high-throughput display formats represents a valuable route. The examples discussed in the scope of this review demonstrate the feasibility of mutant enzyme and scaffold libraries and the inherent potential of directed evolution in combination with the yeast surface display technique for applications beyond common antibody engineering. Yeast display can represent a superior alternative in contrast to other display formats due the utilization of a eukaryotic expression machinery that comprises foldases and chaperones which aid in the folding of scaffold proteins with a demanding three-dimensional fold, such as miniproteins, kringle domains and variable lymphocyte receptors, to name a few. Importantly, yeast display allows a functional readout, another distinct advantage that has been harnessed for protein as well as enzyme engineering with the aim of optimizing catalytic turnover and specificity. However, compared to other display methods the number of variants that can be screened in initial selection rounds is restricted, normally not exceeding 10^8^–10^9^ clones and requires technically demanding and costly equipment, although low-cost alternative cell screening devices may reach the market in the near future [132].

Taken together, yeast display represents a versatile tool for the design and engineering of alternative scaffold proteins and enzymes as various examples have elegantly proven in this context.

## Abbreviations

AgRP: agouti-related protein
ATP: adenosin-triphosphate
CTLA-4: cytotoxic T lymphocyte‐associated antigen 4
CDR: complementarity determining region
EETI: *Ecbacterium elaterium* trypsin inhibitor
EpCAM: epithelial cell adhesion molecule
EphA2: ephrin-type A receptor
FACS: fluorescence-activated cell sorting
FAP: fluorogen-activating proteins
Gp2: T7 phage gene 2 protein
GPI: glycosylphosphatidyl-inositol
HV: hypervariable
IgG: immunoglobulin G
IgNAR: immunoglobulin novel antigen receptor
LRR: leucine-rich repeat
MCoTI: *Momocordia cochinchinensis* trypsin inhibitor
PCR: polymerase chain reaction
SH2: Scr-homology 2
TEV: Tobacco Etch Virus
scFv: single-chain variable fragment
SOTI: *Spinacia oleracea* trypsin inhibitor
VLR: variable lymphocyte receptor
vNAR: variable domain of IgNAR
YSD: yeast surface display
